# X-ray photoelectron spectroscopy of graphitic carbon nanomaterials doped with heteroatoms

**DOI:** 10.3762/bjnano.6.17

**Published:** 2015-01-15

**Authors:** Toma Susi, Thomas Pichler, Paola Ayala

**Affiliations:** 1 University of Vienna, Faculty of Physics, Boltzmanngasse 5, A-1090 Vienna, Austria

**Keywords:** carbon nanotubes, core level photoemission, graphene, substitutional doping, X-ray photoelectron spectroscopy (XPS)

## Abstract

X-ray photoelectron spectroscopy (XPS) is one of the best tools for studying the chemical modification of surfaces, and in particular the distribution and bonding of heteroatom dopants in carbon nanomaterials such as graphene and carbon nanotubes. Although these materials have superb intrinsic properties, these often need to be modified in a controlled way for specific applications. Towards this aim, the most studied dopants are neighbors to carbon in the periodic table, nitrogen and boron, with phosphorus starting to emerge as an interesting new alternative. Hundreds of studies have used XPS for analyzing the concentration and bonding of dopants in various materials. Although the majority of works has concentrated on nitrogen, important work is still ongoing to identify its precise atomic bonding configurations. In general, care should be taken in the preparation of a suitable sample, consideration of the intrinsic photoemission response of the material in question, and the appropriate spectral analysis. If this is not the case, incorrect conclusions can easily be drawn, especially in the assignment of measured binding energies into specific atomic configurations. Starting from the characteristics of pristine materials, this review provides a practical guide for interpreting X-ray photoelectron spectra of doped graphitic carbon nanomaterials, and a reference for their binding energies that are vital for compositional analysis via XPS.

## Introduction

Graphitic carbon nanomaterials consist of carbon bonded via sp^2^-hybridized covalent bonds into structures with dimensionalities in the nanometer scale. Although two naturally occurring forms of carbon, graphite and diamond, have been known for millennia, several new carbon nanomaterials have been created and identified in the last decades. Three recent stages have received major attention, starting with the discovery of fullerenes in the late 1980s [1–2], followed by the proliferation of carbon nanotube research from the early 1990s [3–5], and coming finally to the latest stage when graphene rose into prominence in the mid-2000s [6–8].

**Figure 1:**
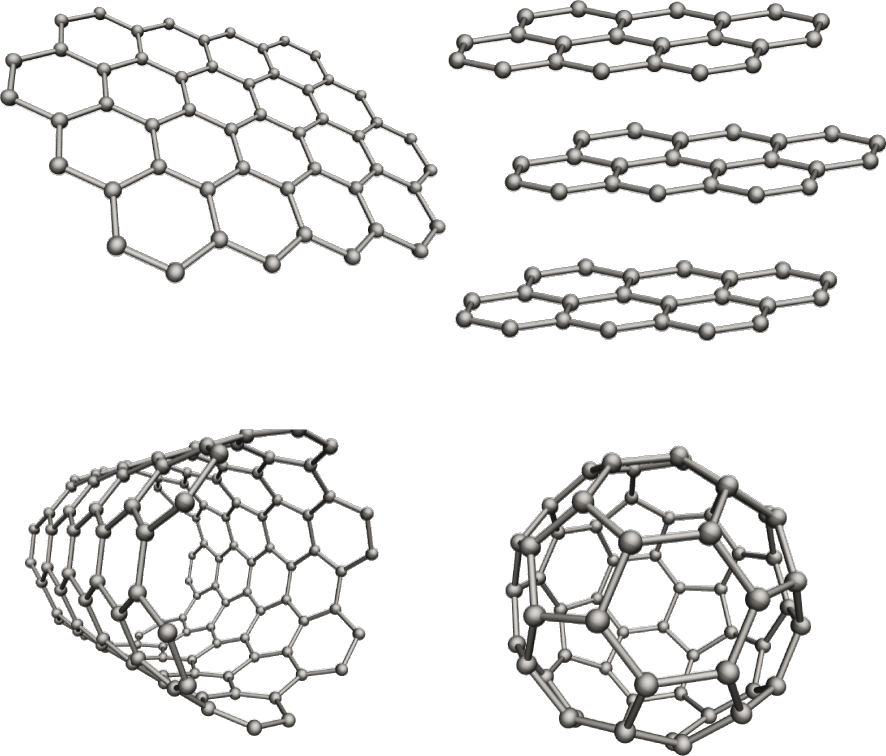
Structural models of graphitic carbon nanomaterials. Clockwise from top left: graphene, graphite, C_60_ fullerene, and a single-walled carbon nanotube.

Due to the unique nature of sp^2^ hybridization [9], strong σ bonds are formed between carbon atoms in fullerenes, nanotubes and graphene (Figure 1), along with delocalized π orbitals [10]. These materials each have superb intrinsic properties. Fullerenes are very stable nanocontainers [11], exhibiting interesting selective surface reactivity [12]. Carbon nanotubes have the highest length-to-diameter ratio of any material, with an extremely high specific strength [13]. Moreover, the single-walled types are either semiconducting or metallic ballistic conductors even at room temperature [14–15], and capable of sustaining current densities 1000 times higher than copper [16–17]. Finally, atomically thin single-layer graphene is extremely elastic yet impermeable [18], and the stiffest and strongest material ever measured [19]. Furthermore, the charge carriers in graphene behave as massless Dirac fermions [7], leading to unparalleled mobility and a number of exotic quantum phenomena [7,20–21].

However, for many real applications, additional control over the intrinsic properties of a material is needed. Heteroatom doping, the intentional replacement of some carbon atoms of the lattice with other elements, has been long studied for this and other purposes [22–23]. One of the key issues for controlled heteroatom doping is the detection and identification of the dopant atoms, and the further analysis of their concentration and atomic bonding environment in the studied materials. Compared to bulk solids, individual nano-objects are composed of far fewer atoms, and thus usual dopant concentrations correspond to a rather limited number of heteroatoms in the lattice. A typical sample must therefore be composed of innumerable such nanostructures in order to reach a measurable quantity, and thus any variability in their properties or in the distribution of dopants poses additional challenges for characterization.

Furthermore, although local methods such as scanning tunneling microscopy (STM) [24–25] and transmission electron microscopy based electron energy loss spectroscopy (TEM/EELS) [23,26] can these days be used to directly visualize and study individual atoms (e.g., [27–32]), it is vital to obtain information about the distribution of the dopants in an entire sample. For this purpose, X-ray photoelectron spectroscopy (XPS) is one of the most attractive techniques, and as such has been widely used to characterize doped materials [33–34]. However, XPS is commonly applied as a laboratory analytical tool, without taking into account important physical factors relevant for carbon nanostructured materials that might affect the resulting analyses. These include, for example, the significant influence of the substrate for measurements of graphene [35–36], and the disentanglement of the intrinsic photoemission responses of semiconducting and metallic single-walled carbon nanotubes (SWCNTs) [37]. Furthermore, as we shall see, identifying the binding energies associated with specific atomic structures is rarely straightforward.

The purpose of this review is to provide a practical guide for interpreting the X-ray photoelectron spectra of doped graphitic carbon nanomaterials, and act as a useful reference for the binding energies that are vital for their compositional analysis via XPS. Angle-resolved photoemission spectroscopy (ARPES), also based on the photoelectric effect [38–39], will only be mentioned briefly in our discussion related to graphene. Graphite has an important role in the context of the carbon 1s line, but our focus will be on graphene and carbon nanotubes, especially single-walled. We will likewise only briefly describe measurement-specific issues and extrinsic effects.

Most importantly, we will only consider heteroatom doping in the lattice itself. This explicitly leaves outside our scope the many forms of functionalization and surface chemistry that have been widely studied [2,12,40–45]. Since the binding energies of even the simplest dopant structures are still being debated, discussion of complicated hybrid structures or doping with more than one element at the same time will be omitted. However, we will endeavor to comprehensively survey the range of binding energy values reported for various configurations to help ensure that the reader has at hand the most complete available evidence for their assignment to specific atomic structures.

## Review

### X-ray photoemission

#### Physical background

We shall divide the X-ray photoemission process conceptually into three stages in a simplified single-particle picture. First, an X-ray photon is absorbed and transfers its energy to a core electron. Then, the target atom responds to this excitation by emitting a photoelectron, creating a core hole, i.e., an empty core state. Finally, the photoelectron transports to the surface of the material and escapes into the vacuum, where its energy is measured by an electron analyzer.

The first step defines to a large degree the binding energy of the core electron (this is called the initial state energy, including any chemical shift characteristic of the material). However, this energy gets modified by the interaction between the photoelectron and the resulting core hole (the so-called final state effects, including many-body interactions and core hole relaxation). Finally, inelastic scattering processes cause some of the photoelectrons to lose kinetic energy on the way to the surface, contributing to a continuous photoemission intensity background (extrinsic effects).

Following the above division of the photoemission process, two types of methodologies can be applied for calculating the core level binding energies: the so-called initial and final state methods [46–52]. In the initial state methods, only the energy level of the core electron before ionization is considered, and shifts in this value between different atoms in the system are calculated. In the final state methods, a core hole is explicitly included in the calculation, and the electronic structure of the system is relaxed in its presence. The core level binding energy is then computed from the total energy difference between a calculation with the core hole and a ground state calculation. More advanced methods aimed at directly simulating the dynamical screening of the core hole and thus the lifetimes and resulting line shapes have also been developed [53–55]. However, since the topic of calculating the core level binding energies in doped carbon nanomaterials is an active area of our research, we will for brevity leave a more detailed discussion of the topic for a later time.

Experimentally speaking, since the carbon core electrons are localized and do not participate in chemical bonding, core level signals could be expected to exhibit very narrow linewidths. However, several factors do cause broadening of the signal and can also influence its shape. Each XPS measurement setup has an intrinsic energy spread of the X-ray source and a finite energy resolution of the analyzer, resulting in so-called instrumental broadening that can be well described by a Gaussian line shape. It is therefore extremely difficult to establish good references when non-monochromatic X-ray sources are used, as is often the case. In addition, a finite sample temperature causes thermal broadening due to phonon vibrations, but this is usually negligible compared to the other factors (since the thermal energy is only approx. 25 meV at 300 K). Finally, each peak has a natural linewidth that is related to the excited state lifetime, usually well described by a Lorentzian profile. Thus, a convolution of Gaussian (G) and Lorentzian (L) profiles, also called the Voigtian (V), is the most adequate way to describe the line shapes *I*_V_(*E*) of the photoemission responses:

[1]
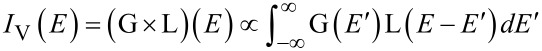


[2]
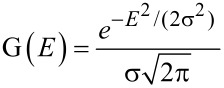


[3]
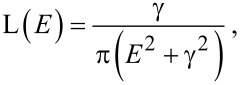


where G(*E*) and L(*E*) are the Gaussian and Lorentzian contributions, σ is the instrumental broadening, and γ the intrinsic lifetime broadening.

However, for a metallic system such as graphite, graphene or metallic carbon nanotubes, in addition to the Gaussian instrumental broadening, the carbon 1s line will exhibit significant asymmetry towards higher binding energies. This is due to excitation of many low-energy electron–hole pairs, which screen the core hole and manifest as higher binding energy events. This asymmetric line shape *I*_DS_(*E*) can be described in the Doniach–Šunjić (DS) [56] form:

[4]
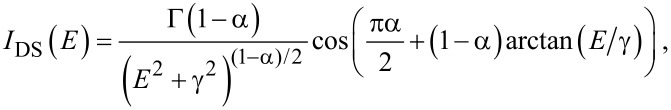


where Γ is the Gamma function


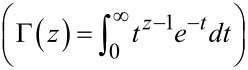


and α is the so-called asymmetry parameter. The asymmetry is zero for semiconductors and insulators, recovering the symmetric Lorentzian form.

#### Measurement

A typical XPS spectrum consists of a so-called primary spectrum, which is a convolution of the final state binding energies of each excited core state, broadened by lifetime and instrumental effects as described above. Superimposed over this is a stepped secondary spectrum, resulting from the inelastically scattered photoelectrons contributing to the higher binding energy sides of each peak. Photoelectrons have a material- and energy-dependent inelastic mean free path λ, which describes their scattering probability before reaching the surface. The sampling depth of XPS is usually taken to be 3λ (i.e., the depth from which 95% of photoelectrons with a normal take-off angle originate; note that 63.3% come from within 1λ), which is between 3 and 10 nm for commonly used Al Kα radiation. While this is well understood for uniform thin films, the sampling depth for nanostructured materials will depend on the sample. For instance, for graphite it is 8.7 nm [57], corresponding to about 26 graphene layers. Thus, for graphitic carbon nanomaterials (apart from thick multiwalled carbon nanotubes) XPS is in effect a bulk-probing technique.

A careful subtraction of the secondary background is vital for a correct analysis of the primary spectrum. Although a number of different background models are in widespread use, a linear background subtraction and the formulations by Shirley [58] and Tougaard [59] are often used.

For evaluating the concentration and the bonding of the dopants, the first sensible step is to focus on the carbon 1s response to evaluate contributions to the spectrum from synthesis byproducts or carbonaceous contaminants. This serves as a prerequisite to further analyze the dopant core states, namely 1s for nitrogen and boron and 2p for phosphorus. Finally, when the background-subtracted areas *a*_X_ of the responses of the sought elements X are multiplied by the corresponding energy-dependent sub-shell photoionisation cross sections σ_x_ [60], their ratios to the area of the carbon response gives the dopant concentration in atomic percent (atom %). As an example, the atomic concentration of nitrogen *c*_N_ would be evaluated as

[5]
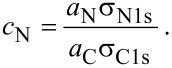


For phosphorus it should be noted that for the commonly considered photoemission from the 2p level, each core state is expected to split into two components due to spin–orbit coupling, corresponding to orbital angular momentum quantum numbers of *j* = 1/2 and 3/2. The magnitude of the spin–orbit splitting is thought to be rather insensitive to the chemical environment and predicted by theory to have a value of 0.87 eV for the P 2p level, with the lower binding energy *j* = 3/2 component having an area twice as large as the *j* = 1/2 component [61]. However, in the studies reported so far, it has not been established whether these predictions directly apply to molecular carbon nanostructures.

A further measurement-related issue is the practice of using the carbon 1s line for calibrating the energy scale, since adventitious carbon often provides a convenient common reference. However, when measuring carbon nanomaterials, the position of the C 1s line should obviously be carefully determined for each sample, and not assumed as a common reference by which the energy scale is calibrated. The absolute position of the C 1s line as well as those signals corresponding to the measured heteroatoms must be calibrated by using a reference. In most cases, a clean metallic sample with a well-understood XPS response is used for this purpose.

### Carbon nanomaterials

In this section, we will briefly introduce the main classes of graphitic carbon nanomaterials, but will refer the reader elsewhere for further information on their properties. Instead, we will concentrate on describing the current state of the art on X-ray photoelectron spectroscopy of each pristine material, including the measured C 1s binding energies and line shapes. In the case of graphite, a consensus has been established in the literature (apart from the existence of a surface state, as discussed below), while for single-walled carbon nanotubes, we have focused on the few measurements made on metallicity-sorted samples, and two representative studies for double-walled and multiwalled nanotubes. For graphene, we have selected studies representing the full range of values measured on various substrates. Table 1 contains an overview.

**Table 1:** Photoemission measurements of the carbon 1s line for various graphitic carbon nanomaterials reported in the literature. The columns show the material, position of the maximum of the carbon 1s response, the energy width of the Lorentzian/Gaussian component, or, the full width at half maximum if these contributions have not been separated, the asymmetry index α, and the literature citation for each measurement.

material	C 1s max. (eV)	L/G width (eV)	FWHM (eV)	asymm. α	references

graphite (G)	284.42	0.16–0.18/0.08–0.1	—	0.05–0.065	[62–64]
G(0001) bulk	284.4^a^	0.095/0.294	—	0.10	[65]
G(0001) surface	284.6^b^	0.231/0.294	—	0.05	[65]
G(0001) bulk	284.4^c^	0.16/0.06	—	0.048	[66]
G(0001) surface	284.52^d^	0.16/0.06	—	0.048	[66]
FLG^e^	284.47	—	0.7	0.1	[67]
SLG/Ni(111)	284.7	0.216	—	0.1	[68]
SLG/Au/Ni(111)	284.2	—	—	—	[69–70]
SLG/Au/Ni(111)	284.42	0.155/0.258	—	0.061	[71]
SLG/Cu	284.5	0.170	—	0.068	[72]
SLG/SiC	284.83	0.12–0.2/0.4–0.7	—	—/0^f^	[64,73–74]
SLG/H/SiC	284.6	—	—	—	[75]
SLG/Pt(111)	283.97	0.13/0.34	—	0.13	[76–77]
4LG/Pt(111)	284.0	—	—	—	[77]
SLG/Ir(111)	284.15	0.130/0.165	—	0.093	[78]
m-SWCNT^g^	284.48	—	0.26	0.11	[37]
s-SWCNT^g^	284.43	—	0.30	0	[37]
(6,5) s-SWCNT	284.52	—	0.41	0	[79]
(6,4) s-SWCNT	284.42	—	0.41	0	[79]
DWCNT	284.6	—	0.64–0.8	0	[80–81]
MWCNT	284.42	—	*>*0.63	—^h^	[82]
C_60_	285.2	0.11/0.60	—	0	[83]

^a^Value not explicitly reported, estimated from graph. ^b^Bulk value not explicitly reported, 195 meV split between the lines attributed to bulk and surface components. ^c^Value not explicitly reported, estimated from graph. ^d^Bulk value not explicitly reported, 120 meV split between the lines attributed to bulk and surface components. ^e^Synchrotron-based scanning X-ray photoelectron microscopy measurements on suspended few-layer graphene. ^f^Either not reported or symmetric line shapes used in the fitting. ^g^Metallicity separated sample with narrow diameter distributions centered at 1.4 nm. ^h^Value not explicitly reported, but remarked to be similar to graphite.

Graphite is a semimetallic solid composed entirely of sp^2^-bonded planar hexagonal carbon layers loosely stacked together by van der Waals interactions. Due to its close kinship to the nanoscale forms, especially graphene, the C 1s line of graphite is the logical starting point for understanding the photoemission response of all carbon nanomaterials. A further advantage specific to highly oriented pyrolytic graphite (HOPG) is that it is relatively straightforward to prepare large but extremely pure and uniform samples.

As mentioned above, in a metallic system such as graphite, the line shape of the C 1s line has the asymmetric Doniach–Šunjić form. The position of the maximum of the peak has been measured at 284.42 eV [62–64,66,84–85], with a lifetime broadening between 160–180 meV and an asymmetry parameter of 0.05–0.065 [62,85]. Some authors have identified another component in the spectra at certain excitation energies or emission angles shifted to higher binding energies by 120–194 meV, and assigned it to the surface layer [65–66]. However, this view has been disputed by others [78,85].

Graphene, a single layer of graphite, is a truly 2-dimensional solid that was originally thought to be fundamentally unstable. When it was experimentally isolated by Geim and Novoselov in 2004 [6], an unprecedented amount of ongoing research activity was launched [8]. The interest was largely due to the unique electronic structure of graphene, whereby the charge carriers behave as massless Dirac fermions [7,86].

The exact position of the intrinsic graphene C 1s line would logically be the one corresponding exclusively to free-standing single-layer graphene. However, this is challenging to measure due to sample-related issues. It is still necessary to understand how the absence of other layers could affect the screening of the core hole and thus cause possible shifts compared to the graphite value. If the assignment of the higher binding energy component discussed above is correct and could be expected to better describe freestanding graphene, this would give it a C 1s value of around 284.6 eV. However, even if this were the case, the influence of the second layer might still have an effect. As we discuss below, in the available measurements on monolayer graphene, it is clear that the role of the surface cannot be discounted.

A recent measurement of graphene on Pt(111) by Rajasekaran et al. [77] found no difference between single- and few-layer graphene (both measured at 284.0 eV). On the other hand, Emtsev et al. [64] and Hibino et al. [73] measured the C 1s energy of mono- to few-layer graphene epitaxial on silicon carbide as a function of the number of layers, and found that the monolayer value is about 0.4 eV higher than the bulk graphite value at 284.8 eV [64,73] (more precisely, 284.83 eV with a lifetime broadening of 0.12–0.2 eV [74]). However, the shift has been attributed to charge transfer from the substrate, similar in magnitude to what has been observed in the Fermi level shift via ARPES [87]. After intercalating hydrogen between the SiC substrate and monolayer graphene, a value of 284.6 eV has been reported [75].

As graphene is commonly grown by chemical vapor deposition on catalytic metals, several XPS measurements on metal surfaces are available. Values of 284.15 [76,78] and 284.2 eV [69–70] have been measured on Ir(111) and Au-intercalated Ni(111) surfaces, respectively. C 1s values for graphene on other metal surfaces range from as low as 283.97 eV on Pt(111) [76–77], to 284.5 eV on Cu(111) [72], and 284.7 eV on Ni(111) [68]. The range of values should make it clear that charge transfer or screening by any particular substrate greatly affects the measured value.

Carbon nanotubes [88] can be conceptually understood as sheets of graphene rolled into seamless cylinders along a certain lattice vector direction, denoted by the so-called chiral indices (*n*,*m*). In single-walled carbon nanotubes, quantum confinement of the electronic wave function around the circumference of the tube results in one third of the tubes being metallic (when *n* − *m* = 3 × integer), while the remaining two thirds are semiconducting with band gaps proportional to their diameter [14,89]. It is also possible that there are several concentric walls approximately separated by the graphite interlayer distance; such tubes are called multiwalled (MWCNTs), with the double-walled (DWCNT) being a somewhat special case [90].

Carbon nanotube samples have two crucial differences compared to graphene in terms of their X-ray photoelectron spectroscopy response. The first difference is an advantage, in that films consisting entirely of self-supported nanotubes (so-called buckypapers) can be made without any substrate. However, it should be noted that depending on the sample preparation, the nanotubes can have a wide range of disorder, diameters, lengths, and degrees of bundling, factors which may all affect the measurement. The second difference, on the other hand, is the complication that samples of SWCNTs typically contain a mixture of semiconducting and metallic tubes, and thus their photoemission response is a convolution of these two different signals. In the past few years, this challenge has been overcome by the development of methods for separating nanotube samples according to their metallicity or even chirality [91–93].

**Figure 2:**
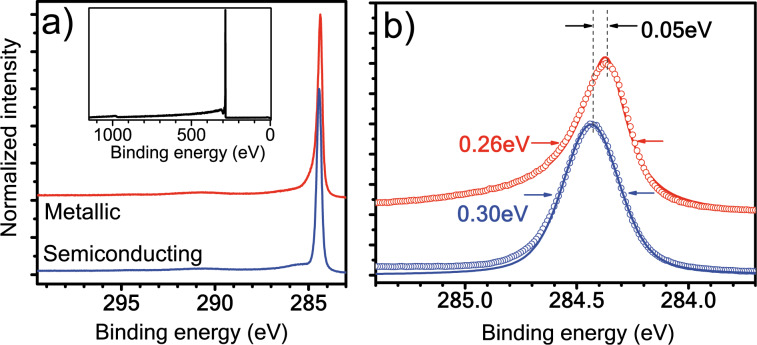
The photoemission response of metallicity-separated and purified single-walled carbon nanotube buckypapers. (a) C 1s photoemission of metallic (red upper curve) and semiconducting (blue lower curve) SWCNTs measured with a 400 eV photon excitation. The inset shows a survey scan, demonstrating the purity of the samples. (b) An expanded view of the C 1s line. The response of the metallic sample has been fitted with a Doniach–Šunjić and the semiconducting with a Voigtian lineshape. Reproduced with permission from [37], copyright 2009 The American Physical Society.

Photoemission measurements from both all-metallic and all-semiconducting SWCNT buckypapers were reported in 2009 [37] (see Figure 2). A further advantage of this study was that both samples had narrow diameter distributions with the same mean, minimizing the variability of properties related to diameter. It was found that the C 1s line of metallic nanotubes exhibits an asymmetric DS shape, with a peak maximum at 284.48 eV and an asymmetry index slightly higher than for graphite at 0.11, and a full width at half maximum (FWHM) of 0.26 eV (narrower than the 0.32 eV found for graphite). On the other hand, the semiconducting sample exhibited a symmetric Voigtian line shape centered at 284.43 eV (FWHM: 0.30 eV). The 0.05 eV difference in the peak position was attributed to differing chemical potentials.

For double-walled carbon nanotubes, a value of 284.6 eV (FWHM: 0.64–0.8 eV) has been reported [80–81]. There is a plethora of measurements on multiwalled nanotubes as surveyed by Schiessling et al., who reported the same position of the C 1s line as for graphite but a larger FWHM at 0.63 eV [82]. However, they attributed this at least partly to remaining disorder or impurities even in the best samples, and cited a number of other measurements on MWCNTs with greater widths even when measured with the same energy resolution.

Although many different fullerene structures are known, the term fullerene (or “buckyball”) often simply refers to the spherical C_60_ molecule resembling a soccer ball, which was the first of their kind to be discovered in 1985 by Kroto et al. [1]. The core level photoemission response of C_60_ is well described by a Voigtian centered at 285.2 eV, with a FWHM of the Lorentzian component measured at 0.11 eV [83].

### Heteroatom doping

After these important considerations of the pristine structures, we turn to our main topic of heteroatom doping. By this we mean the intentional replacement of carbon atoms in the lattice of graphene or the walls of carbon nanotubes by atoms of other elements. Boron (B) and nitrogen (N) are the neighbors of carbon in the periodic table, meaning they have a similar atomic size but one valence electron less (B) or more (N). This makes them the most suitable candidates for heteroatom doping. Individual studies are too numerous to cite here, but a number of reviews are available for general information [33–34,94–95]. Here we will instead only consider the photoemission response of doped carbon nanomaterials.

Although phosphorus (P) was theoretically proposed a long time ago as a possible alternative n-type dopant [96], the first experimental reports on phosphorus doping of CNTs and graphene have only been published recently [97–100]. Like nitrogen, phosphorus has five valence electrons, but since they are on the third electron shell, P has a significantly larger covalent atomic radius (106 pm, compared to 82 pm for B, 77 pm for C, and 75 pm for N), which is expected to cause it to protrude from the graphitic lattice [97,101].

Other heteroatoms such as S, Si, Al and Ni have also been proposed. However, since work in those directions is still rather limited and no XPS data available yet, we will omit them from this review. However, the interested reader can find more information elsewhere [28,102–107].

#### Nitrogen

Nitrogen is undoubtedly the most extensively studied heteroatom dopant for carbon nanomaterials. Stephan et al. pioneered the use of the arc-discharge technique for doping [23], followed by reports on the synthesis of nitrogen-doped multiwalled carbon nanotubes (N-MWCNTs) from several groups [108–110]. Successful nitrogen-doped SWCNT (N-SWCNT) synthesis was reported by Glerup et al. in 2004 by using arc discharge [111], later followed by laser ablation [112] and many different variations of chemical vapor deposition methods [113–125] (see also [33–34]). Nitrogen-doped graphene (N-graphene) has since been synthesized by using numerous methods, amongst them chemical vapor deposition [126–127], post-synthesis treatments [128–129], and ion implantation [30] (see also [107,130]).

Accordingly, there are hundreds of studies that use XPS to study nitrogen doping. However, it does not make sense to try to establish patterns for multiwalled tubes whose photoemission response is in general less well defined [82], and where the internal compartments of N-MWCNTs complicate matters further [131]. Instead, we concentrate on the subset of the literature related to single- and double-walled carbon nanotubes, and for N-MWCNTs and N-graphene restrict ourselves to summarize studies in which synchrotron radiation or an additional complementary technique (such as STM, EELS or X-ray absorption spectroscopy) was used for probing the doping. Table 2 contains our survey, with both the C 1s and N 1s values listed.

**Table 2:** XPS measurements of the carbon 1s and nitrogen 1s lines for various nitrogen-doped graphitic carbon nanomaterials reported in the literature. The columns show the material, diameter of the nanostructure, the carbon 1s energy assigned to C–C bonds, and to C–N bonds, the nitrogen 1s energies assigned to pyridinic (pnic), pyrrolic (plic) and substitutional (subs) nitrogen, the concentration of nitrogen in atomic percent, and the citation for each measurement.

		C 1s (eV)		N 1s (eV)				
material	*d* (nm)	C–C	C–N^a^	pnic	plic	subs	N atomic %	references

(C_59_N)_2_	0.71	285.2	—	—	—	400.72	1.6	[132]
SW	1–1.6	284.5	—	398.5	—	400.6	0.3	[119]
SW	<2	284.8	286.3, 288.3	399.8	—	401.8	3	[121]
SW	1–1.8	284.7^b^	287	397.9	—	401.1	2	[120]
SW	1.1–1.2	—	—	398.6	—	400.5	1.1	[133]
SW	0.9–1.8	—	—	397.6	—	400.5	1	[124]
SW	0.8–1.0	284.5	285.8, 287	398.4	400.9	—	3.2	[134]
S/DW	0.8–2	284.5	—	398.6	—	400.6	0.2	[117]
DW	—	284.5	285.5	398.3	—	400.2	3	[135]
DW	1.6–3.2	284.3	—	398.0	—	401.3	1	[136]
FW	1–5	284.5	287	398.6	—	400.88	6	[116]
MW	15–80	284.5	285.5	398.4	—	400.2	8	[137]
MW	10–40	284.7	285.7 ± 0.1	398.5	—	400.8	4	[138]
MW	30–80	284.5	—	398.6	—	400.5	5	[139]
MW	30–60	284.5	—	398.2	—	400.5	25.7	[140]
MW	20–60	284.1	285.9	398.2	400.2	401.1	5.2	[141]
SLG/Cu	∞	284.8	285.8, 287.5	398.2	400.1	401.7	8.9	[126] ^c^
SLG/Au/Ni(111)^d^	∞	284.4	—	398.4	400.3	401.3	0.48	[127]
SLG/Cu	∞	284.6	285.8	398.6	—	400.6	0.25	[142]
SLG/SiO_2_	∞	284.5	—	398.0	398.9	400	0.4	[143] ^e^
FLG	∞	284.6^f^	—	398.45	399.45	400.92	12.8	[67]
graphite	∞	—	—	398.5	399.9	401.1	2.7	[144]

^a^When two values are listed, the lower binding energy component has been assigned to sp^2^ C–N and the higher to sp^3^ C–N bonds. ^b^Shifted from 284.5 eV upon doping. ^c^Predominant presence of graphitic nitrogen subsequently verified by transmission electron microscopy [145–146]. ^d^Quasi-freestanding graphene via Au intercalation as in [69–70]. ^e^Most values not explicitly reported, estimated from spectrum graph. A slight C 1s downshift and broadening is observed in the doped sample. ^f^Synchrotron-based scanning X-ray photoelectron microscopy measurements on ion-implanted few-layer graphene samples. The pristine sample was measured at 284.47 eV.

In most studies, the C 1s core level of only the doped material is reported, making it difficult to conclude if doping has caused any shift in the binding energy of the bulk system. However, a number of studies have explicitly compared a doped material to a corresponding pristine material [120,128,147–150]. In cases in which comparative data is available, it has been found that the position of the C 1s line is shifted to higher binding energies upon nitrogen doping, with the magnitude of the shift varying from 0.1 up to 0.4 eV depending on the study. Two additional components, most commonly found around 285.8 ± 0.1 and 287.1 ± 0.1 eV, have been tentatively identified and assigned to carbon in sp^2^ C=N and sp^3^ C–N bonds, respectively. However, since higher binding energy contributions could also arise from, e.g., carbon–oxygen bonds, very pure samples would be needed to draw confident conclusions about the identity of such components. Occasionally, values as high as 288.2 ± 0.1 eV have been similarly assigned [121,151], but these seem to be outliers in the literature and perhaps rather attributable to bonds with other elements.

An important and often overlooked starting point for interpreting the N 1s core level are fullerenes containing a single nitrogen substitution, which are called azafullerenes (C_59_N). These are one of the only systems where there is no ambiguity about the underlying atomic structure. Thus even though curvature, the influence of the bonding of azafullerene dimers, or differences in the core hole screening compared to other nitrogen-doped systems may affect the measured value by at most a few hundred meV, the N 1s core level of C_59_N measured at 400.7 eV [132,152] should provide the first estimate for the energy of the N substitution (i.e., graphitic N).

**Figure 3:**
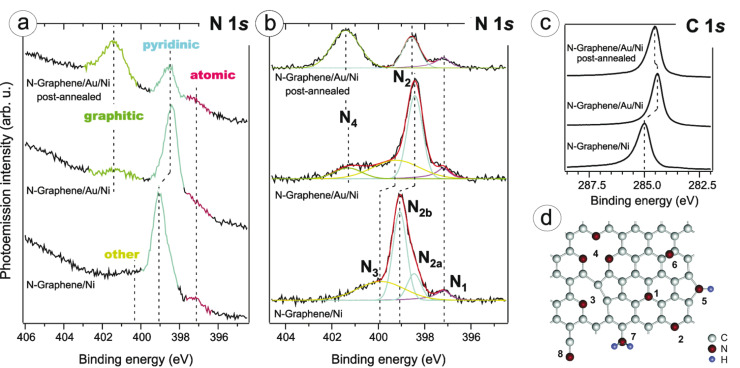
An XPS measurement and peak assignment of nitrogen-doped graphene prepared by chemical vapor deposition. (a–c) N 1s and C 1s spectra of N-graphene freshly prepared on nickel, intercalated by gold, and treated by post-annealing. Note the large shifts caused by interaction with the substrate in the non-intercalated spectrum. The N 1s spectra shown in (b) have been background-subtracted. In the post-annealed sample, a peak at 401.3 eV was assigned to graphitic nitrogen, and another at 398.4 eV to pyridinic N. (d) Some proposed possible configurations of graphene nitrogen impurities: (1) graphitic (substitutional) N; three varieties of pyridinic bonding: (2) edge pyridinic N, (3) single N pyridinic vacancy (1NV), and (4) triple N pyridinic vacancy (3NV); (5) pyrrole-like N, (6) interstitial N or adatom, and (7) amine or (8) nitrile groups. Adapted with permission from [127], copyright 2011 The American Chemical Society.

In general, the N 1s response in XPS has been used for identifying many distinct components in different systems (see Figure 3d for a schematic illustration of many of the different atomic configurations that have been considered). Binding energy values for a N substitution have been variously assigned from around 400.2 eV up to 401.8 eV (e.g., [116,119,121,126–127,132,135,140,149–159]). Even within nominally the same systems (“N-SWCNTs” or “N-graphene”), the spread of the N 1s values is well over 1 eV. There are at least three clear explanations for such a large range of values: inaccuracies in either the measurement or the spectral deconvolution; the assignment of correctly measured binding energies to incorrect atomic configurations; or the effect of differences in core hole screening.

A level of variability could be expected for different systems, and values reported for graphene often tend to be in the higher end of the range. This may be due to the influence of hybridization between the valence orbitals of N with the underlying substrate [32], which reduces core hole screening and thus raises the binding energy, or to an effect of curvature (as proposed for the C 1s line [160]). Figure 3 shows a measurement of N-graphene where a binding energy of 401.3 eV was assigned to the substitutional configuration, and the influence of the substrate clearly elucidated by an intercalation procedure. Perhaps eliminating some of these factors, a very recent measurement using synchrotron-based scanning X-ray photoelectron microscopy assigned a value of 400.92 eV to substitutional N in suspended ion-irradiated few-layer graphene [67]. In any case, the range of values cited for the substitution seems so large that experimental inaccuracies or misassignments likely need to be invoked to explain the variability.

Less controversially, a peak around 398.3 ± 0.3 eV is commonly attributed to pyridinic nitrogen, that is, to nitrogen with a lone electron pair, located either at the edge of the graphitic network or next to a vacancy, and bonded to two carbon atoms [161] (structures 2 and 3, respectively, in Figure 3d). However, occasionally values as low as 397.9 eV [120] and as high as 399.8 eV [121] have been thus assigned. Although this is rarely mentioned, “pyridinic” can implicitly refer to three N atoms surrounding a vacancy (3NV, structure 4 in Figure 3d), or possibly even four N around a divacancy [162]. This is because the formation energy of a single pyridinic vacancy (1NV, structure 3 in Figure 3d) is very high, especially in graphene [163]. While many studies do not find any corresponding local structures in samples where “pyridinic” binding energies are present [164], the 1NV was recently directly observed by STM in ion-implanted graphite [144]; see also [129] for a tentative identification of the 3NV in plasma-treated graphene. Thus it seems the exact atomic configuration of the sites responsible for these peaks still remains somewhat unresolved.

Between these two, binding energies around 400.1 ± 0.3 eV are commonly attributed to “pyrrolic” nitrogen (N in a five-membered ring), which is thought to be responsible for the inner compartments typical for N-MWCNTs [131]. Recently, some authors have suggested that an N substitution in a Stone–Wales defect [165–166] (or otherwise asymmetric local bonding [167]) could also be the origin of this signal. However, it should be noted that amine, pyridone, nitroso and cyano groups could all fall close to this energy [161,168], and thus it is not possible to conclusively determine the exact atomic structure merely based on XPS. Furthermore, considering the N 1s binding energy of C_59_N, it is possible that binding energies at the higher end of this range are incorrectly assigned to pyrrolic N, and are actually graphitic N. If this were the case, however, the atomic origin of the next higher binding energy component around 401.5 eV would have to be rethought (notably, some have attributed this to absorbed N–O instead [143]).

Although not included in our survey in Table 2, components with even higher binding energies between 402 and 405 eV are occasionally also detected. Energies of about 402–403.5 eV have been typically assigned to various oxidized nitrogen configurations ([116,138,141,144,168–170]), pyridine-*N*-oxide being the most widely suggested one [168]). However, binding energies in the same range have also been suggested for clustered N substitutions [67,164,168,171]. Furthermore, hydrogenation of the nitrogen dopants (perhaps of substitutional N, but maybe more plausibly of edge pyridinic N atoms [172–173]) would also be expected to raise the measured binding energies, possibly contributing to the observed overlaps. More studies are needed to elucidate which, if any, of these suggestions play an important role, and in which materials. Finally, values between 404–405 eV (possibly shifted due to physisorption) have been typically assigned to N_2_ molecules trapped inside carbon nanotubes or between graphene layers (e.g., [120,137,140,174–175]).

#### Boron

In boron-doped systems, the identification of the B 1s response is somewhat easier than in the case of nitrogen since a simple substitution is the sole favorable bonding configuration for boron in the lattice [34,176–178]. Very recently, direct local observations of this configuration in graphene were reported by STM [32] and TEM/EELS [30].

The synthesis of boron-doped SWCNTs has mainly been successful through the use of high-temperature techniques, i.e., arc-discharge [23,179] and laser ablation [180]. Identification of dopants was initially mainly via TEM/EELS measurements made directly on the bundles, and as such the presence of elemental boron in the targets or rods was not resolved until a recent XPS study of such a material, in which those bonding environments were clearly discerned [181]. The first successful synthesis of B-doped SWCNTs was carried out by using chemical vapor deposition in a high-vacuum system and a triisopropyl borate precursor [182], and the important role of B adhesion to Fe catalyst particles was also highlighted. For boron-doped graphene, successful synthesis recipes range from the mechanical exfoliation of boron-doped graphite [183] to thermochemical annealing of graphite oxide [184], microwave plasma decomposition of trimethylboron on SiO_2_ [185], and CVD of phenylboronic acid on a copper substrate [186].

It has been consistently reported that the incorporation of B in a nanotube structure is responsible for the formation of an additional lower binding energy peak in the C 1s region [34], which can, depending on the concentration, either appear as broadening of the main peak [181] or as a separate feature [182]. A B 1s response at 187 eV has been assigned to elemental boron, a peak at 189 eV to BC_3_, a peak at 190 eV to B in disordered carbon, and signals between 191.5 and 192.1 eV to substitutional B in the SWCNT wall [181–182]. Higher binding energy components corresponding to boric acid and various boron oxides can sometimes also be found [182], but boron has few gaseous compounds that could attach to the inner walls of nanotubes or adsorb onto the surface of either graphene or nanotubes.

In boron-doped graphene (B-graphene), significantly lower binding energy values from 187 to 189 eV have been assigned to the substitutional B configuration [183–185,187]. At the lower end of the range, Kim et al. [183] assigned a B 1s response of boron-doped graphite at 187.0 eV to substitutional boron. They also saw a downshift of about 0.2 eV of the C 1s line in their doped sample, along with a new peak at 282.2 eV. Sheng et al. [184] similarly measured a downshift of about 0.3 eV of the C 1s line of B-graphene, and assigned B 1s responses at 187.7 and 189.0 eV to B_4_C and B_3_C, respectively; and those at 190.4 and 191.9 eV to BC_2_O and BCO_2_. On the other hand, Tang et al. [185] found a single B 1s response at 189.7 eV, which upshifted to about 190.1 eV with increasing B content. Correlating the XPS measurements by EELS, they assigned this to substitutional boron. Yet higher in energy, Wang et al. [186] measured a single B 1s response at 190.9 eV, again assigning it to substitutional B.

As even the C 1*s* is very sensitive to the substrate on which the measurements are made, determining a fixed value for the binding energy for substitutional boron is not as straightforward in the case of graphene. The effect of the substrate may also be stronger for boron than for nitrogen, since there is evidence that B interacts more strongly with metallic substrates [32].

#### Phosphorus

The production of phosphorus-doped SWCNTs has been reported in a small number of works. Triphenylphosphine [98,188–189], and very recently also trimethylphosphine [190], have been used as P precursors to synthesize P-SWCNTs by using CVD, while Krstič et al. used arc discharge with red phosphorus mixed into the anode rod [99]. The synthesis of P-doped graphene has also been successful to a certain extent, by using ionic liquid 1-butyl-3-methylimidazolium hexafluorophosphate [191]. In addition, the synthesis of P/N-heterodoped graphene has been reported using triphenylphosphine and triphenylamine [100] and by chemical treatment of N-graphene using phosphoric acid [192].

However, unambiguous direct evidence for the bonding of phosphorus in graphitic carbon nanomaterials has been lacking. Based on DFT simulations, it is expected that P will predominantly bond to three C neighbors, but buckle significantly out of the surface due its larger atomic radius [97,101,104]. The curvature in small diameter SWCNTs might make the incorporation of P more favorable by a release of strain [98]. Krstič et al. have further suggested that P substitutions are readily oxidized in ambient, with the P-O bond formation predicted to be exothermic by as much as 3.3 eV [99].

Very limited XPS data is currently available on phosphorus dopants, and the samples used typically contain large amounts of impurities. A P 2p signature around 130.5 eV has been attributed to substitutional phosphorus in P-doped and P/N-co-doped MWCNTs [193–194] and graphene [100]. An additional peak around 132 eV has commonly been attributed to P–O bonds [195–196] (although some authors have instead assigned this peak to the P substitution in purely P-doped graphene [191]). Larrude et al. measured XPS on P-MWCNTs synthesized by spray pyrolysis [197], and assigned two lower binding energy components separated by the expected spin–orbit splitting to substitutional P, with P 2p_3/2_ at 129.3 eV and P 2p_1/2_ at 130.0 eV, with corresponding higher BE components at 133.1 and 133.9 eV assigned to P–O bonds. However, these measurements were performed on an unpurified multiwalled material of relatively poor crystallinity. For P-SWCNTs, the first available measurement found a lower binding energy component at 129.3 eV after annealing purified samples in vacuum, with two higher contributions at 133.3 and 134.2 eV [190].

### Remarks

Considering the literature summarized above, it is clear that large and often overlapping ranges of binding energies has been assigned to allegedly the same atomic configurations. Thus we need to stress that the material in question (e.g., SWCNTs, MWCNTs, graphene on different substrates) must taken into account [37,198], and great care taken when assigning measured binding energies. Careful experiments done under ontrolled conditions using ultrapure materials are still needed to firmly establish reliable signatures for XPS in doped carbon nanomaterials [37]. Furthermore, new theoretical calculations to elucidate the binding energies of various configurations would prove useful, and XPS should be directly correlated with atomically resolved techniques.

With the data at hand, it is interesting to note that in the XPS spectra of nitrogen-doped carbon nanotubes, regardless of the synthesis method there always seems to be at best an approximately equal ratio of binding energy components attributed to pyridinic and graphitic nitrogen, especially in single-walled tubes [115,117,120,124,133]. On the contrary, when graphene crystallinity is high, several studies have reported a clear predominance of the higher binding energy component (e.g., [126–127,142]), often found around 401.5 eV. Although this energy seems rather high to be attributable to (at least single) graphitic nitrogen atoms, local measurements of such samples have seen a clear preference for the simple substitution [27,30,127,142–143,145–146]. It is currently not known whether this difference is due to differences in the formation energies for the various configurations (highly curved structures could favor the formation of pyridinic configurations to release strain energy [162]), or to differences in the morphologies of the respective catalysts, i.e., flat surfaces versus nanosized particles. Further work on elucidating the origin of the differences in the attributed binding energies and their relative abundances in different systems remains greatly needed.

## Conclusion

Significant progress has been made in recent years in synthesizing graphitic carbon nanomaterials doped with various heteroatoms. However, even – or rather, especially – for the most studied dopant, nitrogen, a clear consensus on the core level binding energies and their atomic origin in various systems is still lacking. Furthermore, most of the commonly used binding energy references are still based on measurements made on molecules or by using limited or outdated computational methods, making new theoretical work on the subject desirable in the future. Although studies combining multiple complementary techniques, which thus have the best chances of conclusively identifying the atomic structures, are starting to emerge, much remains to be done to firmly establish the metrology of dopants in carbon nanomaterials.
